# Pulmonary and Functional Rehabilitation Improves Functional Capacity, Pulmonary Function and Respiratory Muscle Strength in Post COVID-19 Patients: Pilot Clinical Trial

**DOI:** 10.3390/ijerph192214899

**Published:** 2022-11-12

**Authors:** Luana Fagherazzi Hockele, João Vitor Sachet Affonso, Danusa Rossi, Bruna Eibel

**Affiliations:** 1 Centro Universitário da Serra Gaúcha (FSG), Caxias do Sul 95020-472, RS, Brazil; 2 Hospital Moinhos de Vento (HMV), Porto Alegre 90035-001, RS, Brazil; 3 Instituto de Cardiologia, Fundação Universitária de Cardiologia (IC/FUC), Porto Alegre 90040-371, RS, Brazil

**Keywords:** COVID-19, pulmonary rehabilitation, functional capacity

## Abstract

Background: Patients affected by COVID-19 may develop an impaired lung function, with reduced lung capacities and volumes, respiratory muscle weakness, changes in radiographic and tomographic findings, limitations in exercising, decreased functional capacity, depression, anxiety and reduced quality of life. Thus, we aimed to analyze the effects of a pulmonary and functional rehabilitation program on the functional capacity, lung function and respiratory muscle strength in patients who were affected by COVID-19 syndrome. Methods: This is a pilot clinical trial, composed of post-COVID-19 patients with mild, moderate or severe involvement, in which, they underwent a pulmonary and functional rehabilitation program. Patients were evaluated for functional capacity by the 6 min walk test, pulmonary function by spirometry, respiratory muscle strength by manovacuometry, handgrip strength by dynamometry, quality of life by the COPD Assessment Test and functional status by the PCFS. After the initial assessments, the patients performed the rehabilitation protocol in 16 sessions (inspiratory muscle training, aerobic exercise and peripheral muscle strength) and, at the end, they were evaluated again. Results: A total of 29 patients completed the program (12.7 ± 2.7 sessions). The functional capacity increased in meters walked from 326.3 ± 140.6 to 445.4 ± 151.1 (*p* < 0.001), with an increase in the predicted value from 59.7% to 82.6% (*p* < 0.001). The lung function increased in liters from 2.9 ± 0.8 to 3.2 ± 0.8 (*p* = 0.004) for forced vital capacity and from 2.5 ± 0.7 to 2.7 ± 0.7 (*p* = 0.001) for forced expiratory volume in the first second. The respiratory muscle strength increased in cmH_2_O from 101.4 ± 46.3 to 115.8 ± 38.3 (*p* = 0.117) for inspiratory pressure and from 85.8 ± 32.8 to 106.7 ± 36.8 (*p* < 0.001) for expiratory pressure. Conclusions: The pulmonary and functional rehabilitation program provided an improvement in the functional capacity, pulmonary function and respiratory muscle strength in post-COVID-19 patients, restoring their quality of life.

## 1. Introduction

The year 2020 began with the news that a new disease had been identified in the city of Wuhan, China [1]. Initially, these were cases of unknown viral pneumonia, which began in a seafood and meat market. It was later discovered that these symptoms came from a bat-derived virus, severe acute respiratory syndrome coronavirus-2 (SARS-CoV-2), designated COVID-19 by the World Health Organization. In a few weeks, the virus spread throughout China, and it did not take long to flood the entire world, causing exorbitant numbers of infections, among many serious cases and thousands of deaths [2]. On 11 March 2020, when the number of infections exceeded the 100,000 mark, the World Health Organization elevated the status of contamination to a pandemic [3].

The transmission of the virus occurs quickly by droplets spreading from an infected person when sneezing, coughing or talking, when they reach the mouth, eyes or nose of people who are close or even by direct contact, such as on a contaminated surface or even in a handshake [4,5].

Studies indicate that most of those infected (81%) have mild symptoms, such as a dry cough, sore throat, fatigue and production of phlegm and fever, and there may also be dyspnea [6], which is characterized by shortness of breath and becomes worrying when there is an increase in the ventilatory drive or work of breathing, with oxygen desaturation. For a minority, particularly those over 65 years of age and with comorbidities such as hypertension, diabetes, asthma, chronic obstructive pulmonary disease, kidney failure and cancer, they may experience more serious consequences, leading to treatment in an intensive care unit setting [6,7].

Patients with severe COVID-19 often develop pneumonia and hypoxemic acute respiratory failure, and many of these patients develop acute respiratory distress syndrome [8]. In addition, other consequences may be present in these patients, such as mechanical-ventilation-induced injury, iatrogenesis by use of steroids and neuromuscular blockers and consequences resulting from immobility, such as atrophy of muscle fibers, blockage and joint pain due to decreased synovial fluid, bone demineralization and pressure ulcers [9]. As a result, patients with COVID-19 may develop an impairment of the lung function, with reduced lung capacities and volumes, respiratory muscle weakness, changes in radiographic and tomographic findings, limitations in exercising, decreased functional capacity, depression, anxiety and reduced quality of life. Many of these sequelae end up impacting the physical, cognitive, mental and social health status, not only in critically ill patients, but also in patients with mild and moderate disease presentation [9,10].

Thus, we aimed to analyze the effects of a pulmonary and functional rehabilitation program on the functional capacity, lung function and respiratory muscle strength in patients who were affected by COVID-19 syndrome.

## 2. Methods

**Figure 1:**
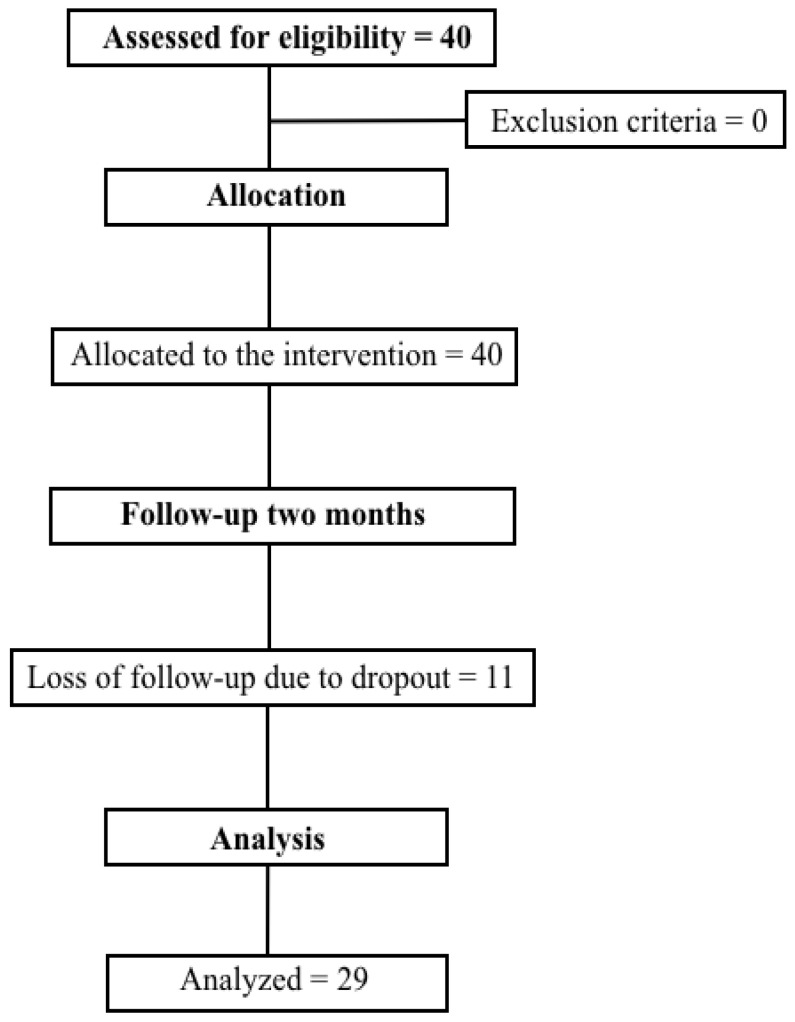
Study flowchart.

**Figure 2:**
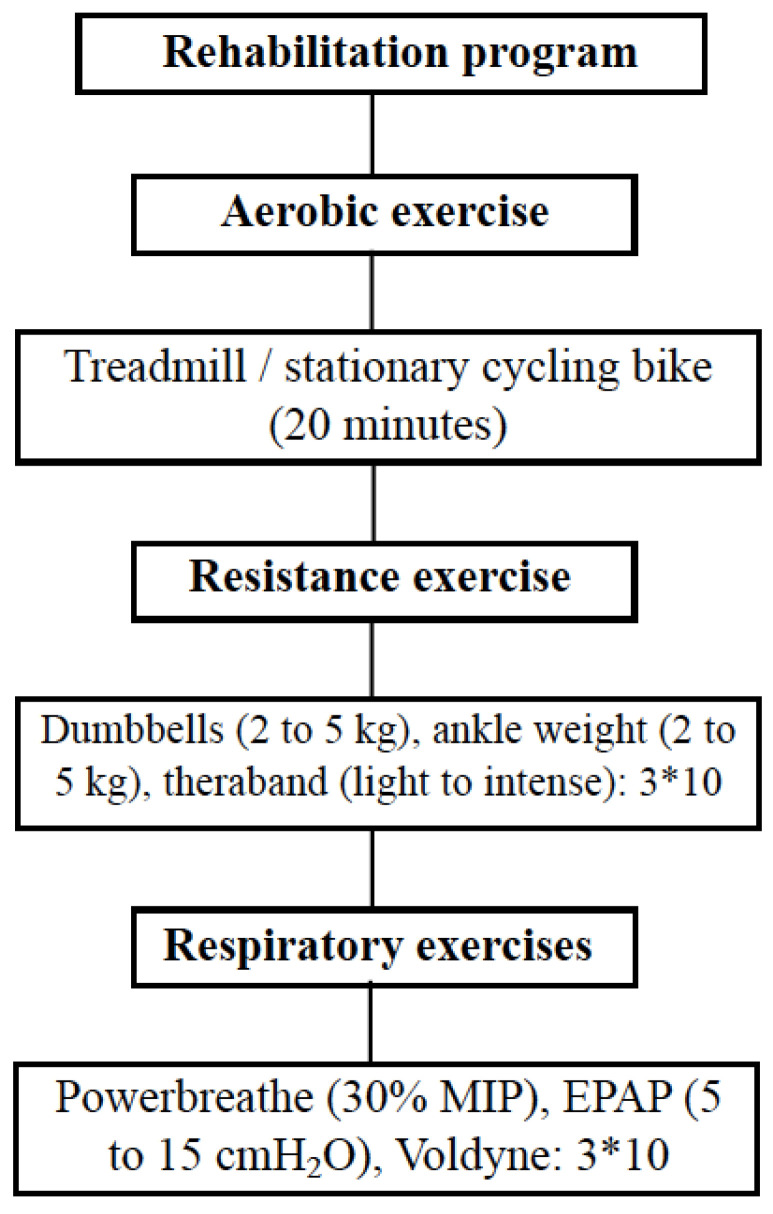
Study protocol. MIP: maximal inspiratory pressure, EPAP: expiratory positive airway pressure.

The present study was a clinical trial, where the sample was composed of post-COVID-19 patients with mild, moderate or severe involvement (defined by chest tomography), where they underwent a post-COVID-19 rehabilitation program. (Figure 1). Patients underwent assessments to assess pulmonary function, respiratory muscle strength, distal muscle strength, functional capacity and functional status. After the tests, the patients performed inspiratory muscle training exercises (IMT), aerobic exercise and peripheral muscle strength exercises, standardized by protocol, but meeting and obeying individual needs and limits (Figure 2), two times per week, for 60 min each. After two months of treatment, the assessments were applied again in order to monitor the evolution of each patient.

### 2.1. Participants

Post-COVID-19 individuals from the list of the Unified Health System of the city of Caxias do Sul, referred by the municipal health department and partner hospitals, were included in the study. The inclusion criteria were participants who were of both sexes and over 18 years of age, with mild, moderate or severe involvement (defined by chest tomography), and who were available to participate in the program twice a week. The exclusion criteria were participants who had cancer or orthopedic limitations. This study was approved by the Research Ethics Committee of the Centro Universitário da Serra Gaúcha—FSG, Caxias do Sul, RS, Brazil. The free and informed consent form was signed by all participants.

### 2.2. Assessments

#### 2.2.1. Manovacuometry

This technique allows for the diagnosis of respiratory failure due to muscle failure and early diagnosis of respiratory muscle weakness, in addition to helping us to assess the response to respiratory muscle training, among many other benefits [11]. To perform the technique, an analog manovacuometer was used (−500 to +500 cm H_2_O), attached to a mouthpiece with an orifice that was two millimeters in diameter. The patient performed the maneuver seated, with the mouthpiece properly positioned and a nose clip, in order to avoid any air leakage during the maneuver [12]. Three measurements were performed for each respiratory phase, the largest being considered verified. The predicted values were based on the formula proposed by Neder et al. (1999) [13].

MIP:

- Women: y= −0.49 (age) + 110.4; estimated standard error = 9.1;
- Men: y= −0.80 (age) + 155.3; estimated standard error = 17.3.

MEP:

- Women: y= −0.61 (age) + 115.6; estimated standard error = 11.2;
- Men: y= −0.81 (age) + 165.3; estimated standard error = 15.6.

#### 2.2.2. Spirometry

Spirometry is a technique used to measure lung function; that is, the amount of air that enters and leaves the lungs, aiding in the diagnosis of ventilatory disorders [14]. For this technique, a MIR spirometer (Rome, Italy) was used: Spirobank II model.

To perform the test, some patient data were collected, such as name, age, sex and anthropometric data. The patient was instructed on the proper positioning during the technique, as well as the execution of the maneuvers and correct use of the clip and mouthpiece [14,15]. A maximum of six maneuvers were performed, with at least three acceptable curves and two reproducible. The expected values as normality were obtained through reference values given by the equipment software [15].

#### 2.2.3. Six-Minute Walk Test (6 MWT)

The six min walk test aims to assess an individual’s submaximal effort when walking for six minutes, and has been widely used in studies with lung and heart disease patients, in addition to predicting mortality and morbidity [16,17]. The test was performed in a 30 m corridor, with markings every three meters, to facilitate the counting of the final distance. The patient was instructed to wear appropriate clothes and shoes during the test, vital signs were measured before and after and oxygen saturation, heart rate and Borg scale were monitored during the test. Patients were instructed to walk as quickly as possible, but not to run or jog, and were allowed to stop if necessary, but to return as soon as possible, as per guidelines from the American Thoracic Society (ATS) [18]. Normality values were based on the formula, published by Soares and Pereira, 2011: 6 MWT = (511+ height^2^ × 0.0066) − (age^2^ × 0.030) − (BMI^2^ × 0.068) [19].

#### 2.2.4. Timed up And Go Test (TUGT)

This test aims to assess functional mobility and risk of falling, as it involves agility, speed and dynamic balance of the user [20]. The test consists of measuring the time, in seconds, that the individual takes to get up from a chair, walk a distance of three meters, turn around, walk back to the chair and sit down again with arms supported. Patients were instructed to wear their normal shoes and their usual walking aids [21]. The normal values were based on those prescribed by Bischoff et al. [22]:

- A total of 10 s or less: fully independent;
- A total of 10 to 19 s: independent for most activities;
- A total of 20 to 29 s: lack of balance and low functional capacity, presenting a moderate risk of falling;
- A total of 30 s or more: Fully dependent for many activities of daily living and at high risk of falling.

#### 2.2.5. Dynamometry

To evaluate the handgrip strength, a hydraulic dynamometer was used. During the test, the individuals were instructed to remain seated, erect, with the limb to be evaluated along the body, the elbow flexed at 90 degrees and forearm in neutral position. Three measurements were taken for each limb, with a rest interval between them, alternating dominant and non-dominant limbs. The highest value recorded for each member was considered [23]. The values found were based on the values described by Massy-Westropp et al., 2004, based on gender, age group and sex [24].

#### 2.2.6. Post-COVID-19 Functional Status (PCFS)

The Post-COVID-19 Functional Status scale was applied in the form of a questionnaire and a flowchart in order to help the patient understand the degree of their limitations in road life. The scale is composed of six stages, where zero represents the absence of functional limitations, and four is severe functional limitations [25].

#### 2.2.7. Modified Medical Research Council (mMRC)

The Modified Medical Research Council scale was used to assess patients’ level of dyspnea in their activities of daily living (ADL). The result can range from zero to four points, where zero is when the patient feels short of breath only during intense exercise, and four is when the patient feels short of breath when leaving the house or getting dressed [26].

#### 2.2.8. COPD Assessment Test (CAT)

The COPD Assessment Test is a questionnaire that measures the impact of chronic obstructive pulmonary disease on the patient’s life, and is composed of eight items (cough, phlegm, chest tightness, shortness of breath when going up or down stairs, limitations in ADL, confidence when leaving home, sleep and energy). Each item received a score from zero to five. The total sum indicated how much the pathology affects the patient’s life, with higher values indicating a worse state of health [27,28].

### 2.3. Statistical Analysis and Sample Size

Data were analyzed using the Statistical Package for the Social Sciences (SPSS) program. Nominal values were presented in absolute and relative frequencies. Parametric data are presented as mean and standard deviation, and the T test was used to compare means. Statistical significance was considered as *p* < 0.05.

To detect a minimum difference of 10% in the improvement of functional capacity, with an error of α = 5%, a power of 80% and a correlation of 0.85, the minimum number of subjects calculated is 42 individuals, based on a study by Camelier et al. (2019) [29], already accounting for possible losses. The program used was WinPepi.

## 3. Results

The sample, composed of twenty-nine patients (Figure 1), had a mean age of 54 ± 14 years, where fifteen of the twenty-nine patients were female (51.7%). The number of sessions, on average, was 12.7 ± 2.7. Regarding the participants, 62.1% were hypertensive; however, other comorbidities were present in the group, including diabetes mellitus (31%), ischemic heart disease (3.4%), hypercholesterolemia (10.3%), cardiac arrhythmia (3.4%), chronic obstructive pulmonary disease (3.4%), asthma (10.3%) and obesity (34.5%), as shown in Table 1. All participants reported being previously sedentary. Among the drug classes used were salbutamol sulfate, budesonide, beta-blocker and metformin.

Table 2 presents data on vital signs, functionality tests, dynamometry and respiratory strength tests, pulmonary function and questionnaires. When analyzing the data, we can see that the vital signs had an improvement in the post-rehabilitation assessment, with a reduction in heart rate (*p* = 0.005), respiratory frequency (*p* = 0.045) and Borg scale (*p* = 0.003), with significantly lower values. In addition, the handgrip strength for the right limb, which is the dominant limb of all patients, had a strength increase (*p* = 0.011), which was also present in the left limb (*p* = 0.006). After the intervention, the twenty-nine patients showed an increase in handgrip strength, and reached the predicted values.

In TUGT, after the intervention, 100% of the patients were classified as totally independent, and the final mean was 10.4 s to perform the test, increasing the speed in seconds significantly (*p* = 0.023).

When analyzing the questionnaires, we can observe that the level of shortness of breath has decreased, and, consequently, the functionality for the ADL has improved. The COPD Assessment Test score went from 15.4 to 8.1 (*p* < 0.001); that is, passing the result of the scale for a great impact of symptoms on the patient’s life reducing to a little impact of symptoms on the patient’s life.

Tests that assess the functional capacity, strength and respiratory muscle function were completed by all participants. The test results are shown in Table 2. After analyzing the data, it was observed that, in the six min walk test, the majority reached the predicted value (82.6%) in the post-intervention evaluation; thus, the distance covered was greater than that performed in the first evaluation (*p* < 0.001).

In the manovacuometry, we can observe that, in the MEP, the sample reached a 129.8% predicted value as normal (*p* < 0.001). In MIP, the values obtained were 138.5% predicted as normal (*p* = 0.088).

In the pulmonary function test, spirometry, we can observe that, in the values referring to the forced expiratory volume in the first second, the participants improved after the rehabilitation program (*p* = 0.001), which was the same when referring to forced vital capacity (*p* = 0.004).

## 4. Discussion

In this pilot clinical trial, in which a pulmonary and functional rehabilitation program was carried out in post-COVID-19 patients, there was an improvement in the functional capacity, proven through the 6 MWT, with an increase in the distance covered, and also in the reduction in the time taken to perform the TUGT. A study carried out in 2013 [30], whose objective was to evaluate the impact of a pulmonary rehabilitation program on patients on the waiting list for lung transplantation, focusing on the quality of life and functional capacity, had a protocol of muscle strengthening, aerobic exercises and multidisciplinary follow-up. Finally, it was possible to conclude that the patients had a significant improvement in the 6 MWT result and in the quality of life, which is in line with our findings.

Our study provided an improvement in the strength of the patients’ respiratory muscles, evidenced by the increase in MIP and MEP, through manovacuometry. Although the MIP results did not reach significant values, there was an increase in the values after the intervention, which can be explained by our sample size. In a randomized study carried out by Winkelmann et al. [31], in which, patients with heart failure and respiratory muscle weakness received aerobic exercise intervention and also IMT, they found a greater statistical significance regarding MIP in patients who received IMT treatment associated with aerobic exercise. The effectiveness of IMT and aerobic exercise was also tested in a study carried out in patients with systemic arterial hypertension, obtaining positive results on blood pressure, functional capacity and the mechanisms of the control of cardiovascular function [32].

In addition, the present study showed an improvement in lung function, confirmed by spirometry, after intervention, in relation to the mean of the predicted values, compared to the pre-intervention assessment. According to Silva et al. 2011 [33], in their research where they evaluated the effects of IMT in hemodialysis patients, spirometry showed no statistically significant difference when comparing the values before and after the intervention. According to the authors, this outcome is due to the fact that IMT aims to improve the inspiratory force, and not lung capacities and volumes. However, we can speculate that our program was able to achieve benefits in both respiratory muscle strength and lung function in post-COVID-19 patients.

In the studied sample, the values for handgrip strength by dynamometry showed an important increase for both limbs. In a study carried out by Costa et al., 2012 [34], it was possible to observe that elderly women, when participating in a group exercise program, had a significant increase in handgrip strength after four months of intervention.

In fact, there was an improvement in the functional status, as well as in the quality of life, confirmed by validated questionnaires, applied before and after the intervention. Studies show that participation in a cardiopulmonary rehabilitation program increases the quality of life of patients with some pulmonary disease [30,35].

In general, we speculate that our protocol, despite the limited sample size and the impossibility of a control group for ethical reasons, corroborates with several studies in the current literature [36,37,38,39], demonstrating the intra-group benefits of a pulmonary and functional rehabilitation program. Similar to our study, Tozato et al., 2021 [16], demonstrated in a series of cases that the four patients had their distance covered during the walk test increase between 16% and 94% and that their peripheral muscle strength was improved by 20% to six times the baseline values, demonstrating that the cardiopulmonary rehabilitation program had a positive impact, improving the functional capacity despite the different severity levels post-COVID-19 cases.

Finally, our study adds to the literature that our number of sessions has benefited the studied sample, and could be extrapolated to other patients affected by COVID-19, optimizing adherence in the follow-up.

## 5. Conclusions

The pulmonary and functional rehabilitation program for post COVID-19 patients, based on aerobic exercises and the strengthening of the respiratory and peripheral muscles, had a positive impact on this series of cases, with an improvement in the functional capacity, pulmonary function, respiratory muscle strength, handgrip strength and, consequently, in ADL. As it is a recent involvement, physical therapy treatment for post COVID-19 patients is still in the process of adaptation.

## Figures and Tables

**Table 1:** Sample characterization (n = 29).

Variables	Frequency (%)
Age (years) ^§^	54.4 ± 14.6
Women *	15/14 (51.7%)
Weight (kg) ^§^	77.4 ± 14.7
Sessions ^§^	12.7 ± 2.7
Comorbidities	
SAH *	18/11 (62.1%)
DM *	9/20 (31%)
Obesity *	10/19 (34.5%)
IHD *	1/28 (3.4%)
Hypercholesterolemia *	3/26 (10.3%)
Cardiac arrhythmia *	1/28 (3.4%)
COPD *	1/28 (3.4%)
Asthma *	3/26 (10.3%)

^§^ Variables described as mean ± standard deviation (SD): age, weight, sessions; * Variables described as frequency: sex, SAH: systemic arterial hypertension, DM: diabetes mellitus, obesity, IHD: ischemic heart disease, hypercholesterolemia, cardiac arrhythmia, COPD: chronic obstructive pulmonary disease, asthma.

**Table 2:** Hemodynamic data on functional capacity, risk of falling, lung function, respiratory muscle strength, dyspnea and functional status.

Variables	Evaluation Pre-Reab	Evaluation Post-Reab	*p*-Value
Vital Signs			
Heart rate (bpm) ^§^	91.7 ± 15.2	84.1 ± 12.9	0.005 *
Respiratory rate (rpm) ^§^	21.1 ± 5.1	19.3 ± 4.7	0.045 *
Oxygen saturation (%) ^§^	96.1 ± 2.6	96.2 ± 2.4	1.000
Blood Pressure			
Systolic (mmHg) ^§^	131.7 ± 15.3	128.6 ± 14.8	0.272
Diastolic (mmHg) ^§^	88.2 ± 10.7	85.2 ± 17.7	0.432
Borg ^§^	2.6 ± 2.1	0.8 ± 1.1	0.003 *
Functional tests			
TUGT (s) ^§^	13.9 ± 9.1	10.4 ± 6.3	0.023 *
6MWT (m) ^§^	326.3 ± 140.6	445.4 ± 151.1	<0.001 *
6MWT (%) ^¥^	59.7 ± 23.8	82.6 ± 28.2	<0.001 *
Manovacuometry			
MIP (cmH_2_O) ^§^	101.4 ± 46.3	115.8 ± 38.3	0.117
MIP (%) ^¥^	120.0 ± 49.7	138.5 ± 44.6	0.088
MEP (cmH_2_O) ^§^	85.8 ± 32.8	106.7 ± 36.8	<0.001 *
MEP (%) ^¥^	104.3 ± 37.1	129.8 ± 41.3	<0.001 *
Dynamometry			
Right (Kg) ^§^	28.6 ± 21.0	37.4 ± 19.6	0.011 *
Left (Kg) ^§^	26.6 ± 20.3	35.4 ± 18.7	0.006 *
Spirometry			
FVC (L) ^§^	2.9 ± 0.8	3.2 ± 0.8	0.004 *
FVC (%) ^¥^	3.7 ± 0.9	3.8 ± 0.8	0.412
FEV_1_ (L) ^§^	2.5 ± 0.7	2.7 ± 0.7	0.001 *
FEV_1_ (%) ^¥^	2.9 ± 0.7	3.0 ± 0.7	0.427
FEV_1_/FVC (%) ^¥^	80.1 ± 2.4	80.2 ± 2.3	0.770
Questionnaires			
mMRC ^§^	1.79 ± 1.0	0.68 ± 0.7	<0.001 *
CAT ^§^	15.4 ± 8.6	8.1 ± 7.3	<0.001 *
PCFS-A ^§^	2.1 ± 1.4	1.0 ± 1.2	<0.001 *
PCFS-B ^§^	2.0 ± 1.4	1.0 ± 1.1	<0.001 *

Reab: rehabilitation; rpm: respirations per minute; bpm: beats per minute; mmHg: millimeters of mercury; sec: seconds; kg: kilogram; ^§^ Variables described as mean ± standard deviation (SD): heart rate, respiratory rate, saturation, systolic, diastolic, Borg, TUGT: timed up and go test, 6 MWT: 6 min walk test, MIP: maximal inspiratory pressure, MEP: maximal expiratory pressure, right and left dynamometry, FVC: forced vital capacity, FEV_1_: forced expiratory volume in one second, mMRC: Modified Medical Research Council, CAT: COPD Assessment Test, PCFS-A: Post-COVID-19 Functional Status-A, PCFS-B: Post-COVID-19 Functional Status-B; ^¥^ Variables described as the mean of the ratio between the predicted and the achieved value ± standard deviation (SD): 6 MWT, MIP, MEP, FVC, FEV_1_, FEV_1_/FVC: ratio between expiratory volume in one second and forced vital capacity; * *p* value < 0.05.

## Data Availability

Not applicable.
